# Echovirus 6 Infects Human Exocrine and Endocrine Pancreatic Cells and Induces Pro-Inflammatory Innate Immune Response

**DOI:** 10.3390/v9020025

**Published:** 2017-01-30

**Authors:** Luis Sarmiento, Gun Frisk, Mahesh Anagandula, Monika Hodik, Ilaria Barchetta, Eitan Netanyah, Eduardo Cabrera-Rode, Corrado M. Cilio

**Affiliations:** 1 Cellular Autoimmunity Unit, Department of Clinical Sciences, Skåne University Hospital, Lund University, Jan Waldenströms gata 35, CRC 91:10 205 02 Malmö, Sweden; ilariabarchetta@hotmail.com (I.B.); eitan.netanyah@med.lu.se (E.N.); corrado.cilio@med.lu.se (C.M.C.); 2 Department of Immunology, Genetics and Pathology, Uppsala University, Rudbeck Laboratory, 75185 Uppsala, Sweden; gun.ergida.frisk@gmail.com (G.F.); mahesh.anagandla@gmail.com (M.A.); monika80hodik@gmail.com (M.H.); 3 Department of Internal Medicine and Medical Specialties, Sapienza University of Rome, 00185 Rome, Italy; 4 Department of Immunology and Genetics on Diabetes, National Institute of Endocrinology, 10400 Havana, Cuba; diabetes@infomed.sld.cu

**Keywords:** acinar cells, echovirus, enterovirus, inflammation, islet of Langerhans, pancreas, tropism

## Abstract

Human enteroviruses (HEV), especially coxsackievirus serotype B (CVB) and echovirus (E), have been associated with diseases of both the exocrine and endocrine pancreas, but so far evidence on HEV infection in human pancreas has been reported only in islets and ductal cells. This study aimed to investigate the capability of echovirus strains to infect human exocrine and endocrine pancreatic cells. Infection of explanted human islets and exocrine cells with seven field strains of E6 caused cytopathic effect, virus titer increase and production of HEV protein VP1 in both cell types. Virus particles were found in islets and acinar cells infected with E6. No cytopathic effect or infectious progeny production was observed in exocrine cells exposed to the beta cell-tropic strains of E16 and E30. Endocrine cells responded to E6, E16 and E30 by upregulating the transcription of interferon-induced with helicase C domain 1 (IF1H1), 2′-5′-oligoadenylate synthetase 1 (OAS1), interferon-β (IFN-β), chemokine (C–X–C motif) ligand 10 (CXCL10) and chemokine (C–C motif) ligand 5 (CCL5). Echovirus 6, but not E16 or E30, led to increased transcription of these genes in exocrine cells. These data demonstrate for the first time that human exocrine cells represent a target for E6 infection and suggest that certain HEV serotypes can replicate in human pancreatic exocrine cells, while the pancreatic endocrine cells are permissive to a wider range of HEV.

## 1. Introduction

Human enteroviruses (HEV), in particular the coxsackievirus serotypes B (CVB) and echoviruses (E), have long been linked to diseases of both the exocrine and endocrine pancreas such as pancreatitis and type 1 diabetes, respectively [1,2]. Several independent studies have shown that members of both CVB and E replicate in explanted human pancreatic islets, causing functional beta cells impairment and local virus-induced production of pro-inflammatory cytokines and chemokines [3,4,5,6]. Thus, the exposure to virus strains targeting beta cells and inducing innate and adaptive immune responses may represent the mechanism through which HEV infection is involved in the pathogenesis of type 1 diabetes. 

Despite the well-documented tropism of HEV toward human pancreatic islets, little is known about the human exocrine pancreas as a HEV target. To date, there is no direct evidence of HEV infection in the exocrine component of the pancreas. Evidence supporting the concept of a HEV etiology in the development of pancreatitis derives from serological studies [7,8] and case reports [9,10,11]. However, the assumption of exocrine infection by HEV in humans has not been confirmed by ex vivo studies. 

Animal studies have shown that CVB replicates to high titers in mouse pancreas with subsequent acinar cell destruction, immune infiltration [12], and severe acinar pancreatitis [13]. Furthermore, virus particles, HEV capsid protein VP1 and RNA of CVB have been identified in acinar cells by electron microscopy [14], immunohistochemistry [15] and in situ hybridization [16]. In contrast with data in mice, studies on pancreases from patients with type 1 diabetes demonstrated the presence of HEV-positive cells in pancreatic islets whereas neither CVB-specific hybridization signal nor HEV genomes and HEV capsid protein VP1 were detectable in exocrine cells [3,17,18]. Likewise, tropism analysis of two CVB5 strains revealed virus growth in human explanted pancreatic islets but not in exocrine cell clusters, supporting the overall view that, in humans, CVB tropism in vitro is restricted to pancreatic endocrine cells [19].

However, most of the aforementioned studies have focused mainly on CVB, and there has been no attempt to assess the possible tropism of echovirus towards exocrine cells. Although we previously demonstrated that strains of E16 and E30 with a proven association with islet autoimmunity were able to replicate in explanted human islets and β cell-derived lines [20,21,22], their effect on human exocrine cells has never been tested. Interestingly, maternal infection with E6, a closely related echovirus strain, was associated with the induction of coexisting neonatal type 1 diabetes and exocrine pancreatic insufficiency in a Finnish report [23]. Further evidence supporting the idea that E6 may induce exocrine pancreatic damage in humans is the finding of significantly risen antibodies titers against E6 in sera from patients with acute pancreatitis [8]. Thus, the existence of specific HEV with a tropism for both human exocrine and endocrine pancreas cannot be completely ruled out.

Therefore, the aims of this study were to investigate the capability of E6 to infect explanted human exocrine and endocrine pancreatic cells and to assess the tropism of E16 and E30 toward human exocrine cells. This study also evaluated the virus-induced expression of innate immunity genes in both exocrine and endocrine pancreatic cells and examined to what extent the virus infection affects the expression of the genes encoding amylase and insulin.

## 2. Material and Methods

### 2.1. Viruses

For these purposes, we selected seven field strains of E6 isolated from the stools of sporadic cases of viral meningitis in Cuba during the years 1991 (E6/91), 1992 (E6/92), 1993 (E6/93), 1994 (E6/94), 1996 (E6/96), 2011(E6/11) and 2012 (E6/12) [24]. Clinical strains of E16 and E30 isolated during the Cuban meningitis epidemic in the years 2000 and 2001, respectively, from stool of subjects that developed islet cells antibodies during the convalescence stage of the infection were also included in the study [25,26]. Stool samples from the subjects were collected and processed according to standard procedures recommended by the World Health Organization (WHO) [27].

All viruses were propagated on 90% confluent green monkey kidney (GMK) cells in Eagle’s minimum essential medium (EMEM) supplemented with 10% (v/v) fetal bovine serum (FBS), 100 u/mL penicillin and 0.1 mg/mL streptomycin. Crude virus preparations were clarified from cell debris by centrifugation at 400 *g* for 10 min. The viral titer was measured by the cytopathic effect in microtitration assays and expressed as a 50% cell culture infectious dose (CCID50) per mL according to the Kärber formula [28]. The identity of all the isolates was confirmed by neutralization tests with type-specific antisera and partial VP1 sequences by primer pairs 187 (VP1; 5′-ACIGCIGYIGARACIGGNCA-3′) and 011 (2A; 5′-GCICCIGAYTGITGICCRAA-3′) (Thermo Fisher Scientific Waltham, MA, USA) and comparison with published sequences [29].

### 2.2. Tissue Source and Purity

Human pancreases from 14 organ donors free of any pancreatic disease were obtained from the Nordic Network for Islets Transplantation, Uppsala University, Uppsala, Sweden and human tissue laboratory of Lund University Diabetes Center, Malmö, Sweden [30], using a protocol described elsewhere [31]. For our purposes, we were able to obtain pancreatic islets from four donors, exocrine cell clusters from seven donors and both islets and exocrine cell clusters from the remaining three donors. Islets and exocrine cell clusters were kept in culture bags (Baxter Medical AB, Kista, Sweden) with 200 mL CMRL-1066 (ICN Biomedicals, Costa Mesa, CA, USA) supplemented with 10 mM 4-(2-hydroxyethyl)-1-piperazineethanesulfonic acid (HEPES), 2 mM l-glutamine, 50 mg/mL gentamycin, 0.25 mg/mL fungizone (Gibco BRL; Invitrogen Ltd., Paisley, UK), 20 mg/mL ciprofloxacin (Bayer Healthcare AG, Leverkusen, Germany), and 10% heat-inactivated human serum at 37 °C in 5% CO_2_ and humidified air for 1–7 days.

In order to obtain a highly purified population for each cell type, islets and exocrine cell clusters were further handpicked with a micropipette under an inverted light microscope. The isolated and handpicked cell preparations used were highly pure (>95%) as measured by dithizone and insulin staining. All islets were intensely stained, whereas the exocrine cells displayed just focal and weak staining in few sections. All experiments were performed on 70 islets and 70 acinar cluster per well, and cultured in non-attach six-well plates (Sarstedt, Numbrecht, Germany) in 2 mL Roswell Park Memorial Institute (RPMI) medium containing 5.5 mM glucose (SVA, Uppsala, Sweden), supplemented with 10% FBS and 2 mM l-glutamine.

### 2.3. Virus Replication and Cytopathic Effect

Free-floating exocrine cell clusters and islets from each donor were divided into parallel aliquots and either infected with a 1000 CCID50/0.2 mL of each viral strain or mock-infected as a control. All the cells were incubated at 37 °C and examined under a light microscope for a period of 3–5 days, depending of the appearance of cytopathic effect/islet dissociation or not. Virus replication was determined by CCID50 titration method. Briefly, 0.2 mL of 10-fold serial dilutions (1:10 to 1:10^8^) of samples of the culture medium collected on day 0, and day 3 post infection, were added in triplicate to GMK cells cultured in 96-well plates. Cytopathic effect was read on day 5 and CCID50 titer was calculated using the Kärber formula [28]. The virus replication extent was expressed as the difference between the CCID50 titer at day 3 and at 0 day post-infection (samples of culture medium collected directly after infection).

### 2.4. Gene Expression

Isolated human pancreatic islets and exocrine cells from three donors were washed in phosphate-buffered saline (PBS) and lysed by using RLT buffer (Qiagen, Sollentuna, Sweden) on QIAshredder spin columns (Qiagen). Total RNA was extracted with RNAeasy Mini kit. RNA quantity and quality were determined by Nanodrop (Thermo Scientific, Braunschwig, Germany). Fifty nanograms of total RNA per sample were primed with random hexamer and reverse transcribed to cDNA with SuperScript IITM RT (Invitrogen) according to the manufacturer’s instructions. The reaction was carried out at 25 °C for 10 min, 42 °C for 55 min and for 15 min at 75 °C. Real-time PCRs were run with SYBER Green master mix (Applied Biosystems, Stockholm, Sweden) in a 96-well optical plate on a StepOnePlusTM Real-Time PCR system (Applied Biosystems). The reaction mixture consisting of 10 µL Syber Green master mix (Applied Biosystems), 2 µL primer and 1 µL cDNA was prepared according to the manufactures instructions. The cycling conditions were 40 cycles of 15 s at 94 °C, 30 s at 55 °C and 30 s at 68 °C. Predesigned genes specific primer sets (QuantiTect1 Primer Assays; Qiagen) were used for detecting the expression of interferon induced with helicase C domain 1 (IF1H1), 2′–5′-oligoadenylate synthetase 1 (OAS1), interferon-β (IFN-β), chemokine (C–X–C motif) ligand 10 (CXCL10), chemokine (C–C motif) ligand 5 (CCL5), insulin and amylase. The mRNA expression of each gene was normalized to the expression level of 18S and β-actin housekeeping gene using Δ*C*_t_ method and is presented as 2^−Δ*C*t^ values, where *C*t is the cycle threshold. The gene expression of infected cells was compared with the one of the mock-infected cells. Melt curve analysis was used to verify the specificity of final PCR products.

### 2.5. Glucose Stimulation Test

Fifty hand-picked and size-matched islets from three donors were infected with E6 strains isolated in 1991 and 2012 or mock-infected as a control. Insulin secretion in response to glucose stimulation was assessed on day 3 of culture in a dynamic perifusion system (Suprafusion 1000; BRANDEL, Gaithersburg, MD, USA). Islets were perifused with two glucose concentrations (1.67 and 16.7 mmol/L and then 1.67 mmol/L again), fractions were collected at six-minute intervals for 120 min and insulin concentration was determined by enzyme-linked immunosorbent assay (ELISA) (Mercodia, Uppsala, Sweden).

### 2.6. Immunohistochemistry

Explanted islets and exocrine cell clusters of three donors were either mock-infected or infected with the E6/11 strain. Viruses-infected and mock-infected islets and exocrine cells were transferred to glass tubes on day 3 post infection. They were washed in PBS, fixed in 4% buffered paraformaldehyde (PFA), dehydrated in graded ethanol and embedded in paraffin. The paraffin embedded cells were sectioned (5 mm) and dried on Superfrost glass slides (Menzel-Gläzer; Fischer Scientific, Braunschweig, Germany), followed by de-paraffinization and re-hydration in 99%–70% ethanol. Antigen retrieval was performed in pH 9 TE buffer (10 mM Tris-HCl, 1 mM ethylenediaminetetraacetic acid (EDTA); DAKO, Glostrup, Denmark) in a steam boiler and permeabilized in PBS containing 0.05% TWEEN 20 (Sigma–Aldrich, St. Louis, MO, USA). Endogenous peroxidase was blocked by the use of a ready-to use peroxidase blocker (DAKO). After rinsing with PBS for 10 min, they were stained at room temperature with monoclonal antibody against a broad reacting epitope on the structural HEV protein 1 (VP1). Incubation with this antibody was performed at room temperature for one hour and the visualization was achieved by the anti-mouse Envision-kit (DAKO), using 3,3′-diaminobenzidine (DAB) as substrate chromogen.

### 2.7. Electron Microscopy

Islets and exocrine cells from a representative donor were infected with the E6/11 strain. Virus-infected and mock-infected samples were fixed in 2% glutaraldehyde in 0.1 M cacodylate buffer supplemented with 0.1 M sucrose, followed by 1.5 h post fixation in 1% OsO4, dehydration and embedding in epoxy plastic agar 100 (Agar Aids, Ltd., Stansted, UK). Ultra-thin sections (50 nm) were placed on Formvar-coated Cu grids (Agar Aids, Ltd., Stansted, UK), contrasted with uranyl acetate and lead citrate and analyzed in a Tecnai 12 BIO Twin electron microscope (FEI Company, Hillsboro, OR, USA).

### 2.8. Statistical Analysis

All statistical analyses were performed using SPSS package version 23 (IBM Corp., Armonk, NY, USA). Data are presented as mean value ± standard deviation (SD). All the experiments included control and virus-infected samples from the same donor. Comparisons among multiple groups were evaluated by Bonferroni-adjusted one-way ANOVA test. Mann-Whitney U test was used for comparisons between two groups. A *p*-value <0.05 was considered as statistically significant, with a confidence interval of 95%.

### 2.9. Ethics Statement

The study was conducted in compliance with the principles expressed in the Declaration of Helsinki and in the European Council’s Convention on Human Rights and Biomedicine. All methods were carried out in accordance with relevant guidelines and regulations. Consent for organ donation (for clinical transplantation and for use in research) was obtained from the relatives of the deceased donors by the donor’s physicians and documented in the medical records of the deceased donor. All procedures were approved by ethics committees at Uppsala University (Permit number: Dnr 2009/043, 2009/371, Ups 02-577) and Lund University (Permit number: Dnr.173/207). Consents for the use of the stool samples were waived because only left-over samples were used.

## 3. Results

### 3.1. Primary Human Pancreatic Exocrine and Endocrine Cells Are Productively Infected by Echovirus 6

**Figure 1:**
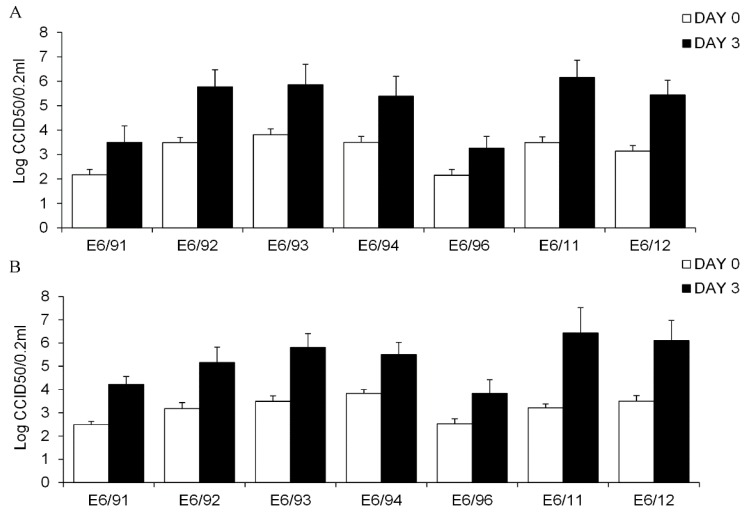
Replication of field strains of echovirus 6 (E6) in primary human exocrine cells (**A**) and into the islets (**B**). Free-floating human exocrine cells derived from ten donors and islets from seven donors were infected with a 1000 50% cell culture infectious dose (CCID50)/0.2 mL of E6/91, E6/92, E6/93, E6/94, E6/96, E6/11 and E6/12. The samples were taken at day 0 (empty bar) and day 3 (filled bar) post-infections and were assayed for total infectivity by using the CCID50 titration method. Data are presented as log10 (CCID50/0.2 mL), means ± standard deviation (SD) from experiment performed in triplicate.

The infection with the seven different strains of E6 isolated during a period of 21 years, resulted in increased viral titer in both explanted human exocrine and endocrine cells after 72 h (Figure 1). In exocrine cells, the increase of virus titer during the three days post-infection ranged from 1.12 (E6/96) to 2.70 (E6/11) log10 CCID50/0.2 mL, 1.86 ± 0.59; mean ± SD (Figure 1A) whereas for endocrine cells ranged from 1.33 (E6/96) to 3.30 (E6/11) log10 CCID50/0.2 mL, 2.20 ± 0.64 mean ± SD (Figure 1B).

**Figure 2:**
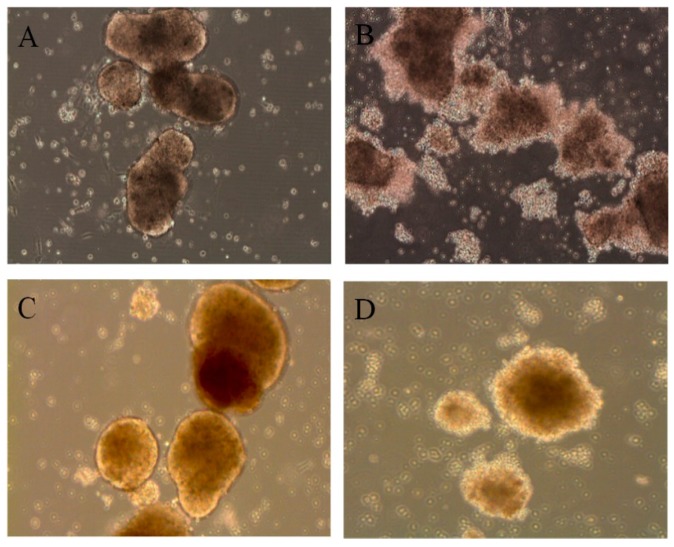
Virus-induced cytopathic effect in explanted human pancreatic exocrine cells and islets three days post-infection. (**A**) Mock-infected exocrine cells; (**B**) E6-infected exocrine cells; (**C**) Mock-infected islets; (**D**) E6-infected islets. The present results are a sample from different experiments with similar findings performed on islets from seven donors and exocrine cells from ten donors inoculated with the seven strains of E6.

Bonferroni-adjusted ANOVA test for multiple comparisons showed that, among all the E6 strains, E6/11 infection resulted in significantly higher viral titer in exocrine (*p* = 0.006) and endocrine (*p* = 0.001) cells in comparison with that observed after the infection with the other E6 strains. However, the overall difference in virus titer increase was not statistically significant between islets and exocrine cells (*p* = 0.146). All the E6 strains induced similar morphologic alterations in exocrine (Figure 2A,B) and endocrine cells (Figure 2C,D), with cytopathic effects detectable 72 h post-infection.

**Figure 3:**
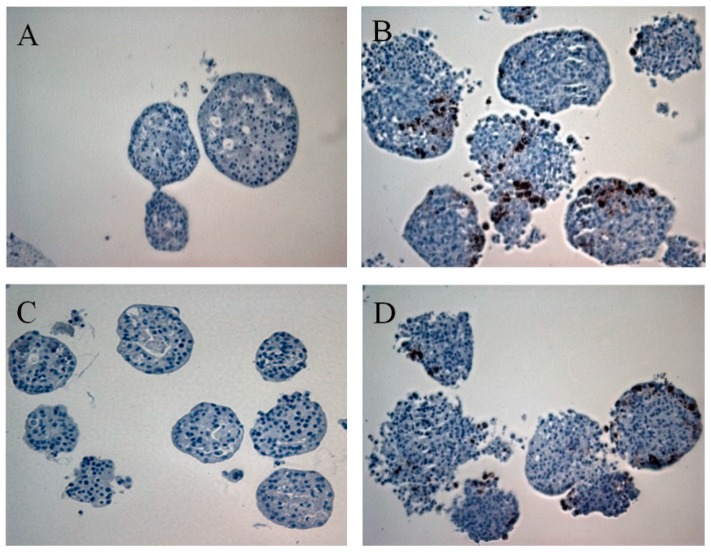
Immunostaining for human enteroviruses (HEV) protein VP1 in exocrine cells and islets three days after infection with E6/11. (**A**) Mock-infected exocrine cells; (**B**) E6-infected exocrine cells; (**C**) Mock-infected islets; (**D**) E6-infected islets. Representative example of three independent experiments.

**Figure 4:**
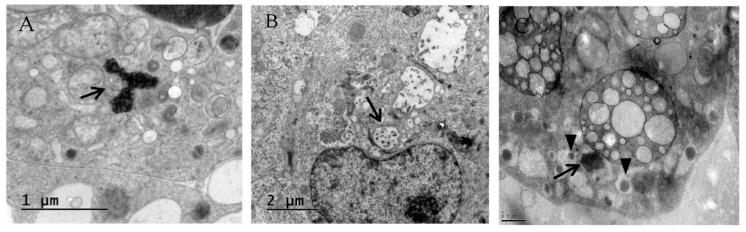
Electron microscopy of exocrine and endocrine cells infected with E6/11. (**A**) Virus particles (arrow) arranged in arrays in the cytoplasm of acinar cells, scale bar: 1 μm; (**B**) Portion of acinus showing degraded cytoplasm, membrane vesicles and vacuoles (arrow), scale bar: 2 μm; (**C**) Beta cells from islets infected with E6 containing virus particles arranged in a cluster (arrow) in close proximity to insulin granules (arrowhead), scale bar: 0.5 μm.

**Figure 5:**
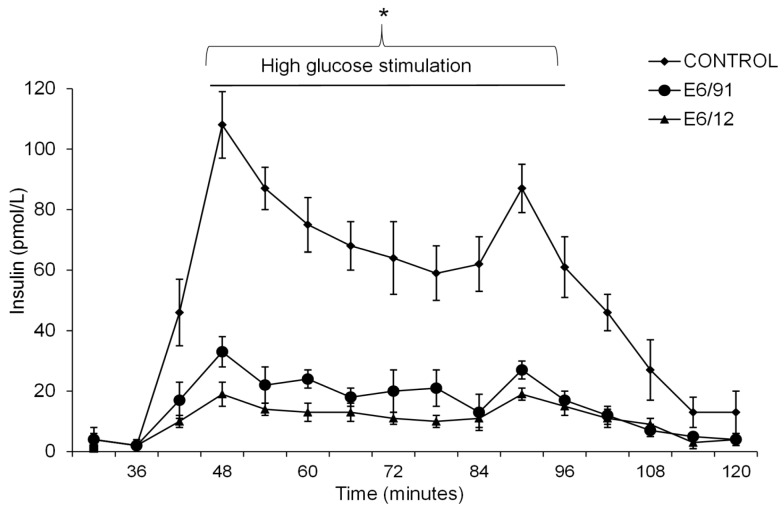
Dynamic release of glucose-stimulated insulin secretion in E6-infected islets. Fifty hand-picked and size-matched islets from three donors were infected with E6/91 and E6/12. Mock-infected islets were left as a negative control. On day three after infection, the islets were perifused with glucose (1.67 mmol/L, 16.7 mmol/L, and 1.67 mmol/L). Fractions were collected at six-minute intervals and the secreted insulin was measured by enzyme-linked immunosorbent assay (ELISA). Data are presented as means ± SD and are based on observations from at least three donors. * *p* < 0.05 between groups (minutes 42 to 102).

Acinar (Figure 3A,B) and islet (Figure 3C,D) cells infected with E6/11 strain were stained positive with antibody directed against the HEV capsid protein VP1. Virus particles were observed by electron microscopy in exocrine and endocrine cells infected with E6/11 strain (Figure 4). Aggregates of virus and membrane-bound vesicles (Figure 4A) were detected in the exocrine cell cytoplasm. Echovirus 6-infected acinar cells showed a massive degradation of cytoplasm along with an increased number of vesicular structures and vacuoles containing virus-like particles (Figure 4B). Echovirus 6-infected islets exhibited an accumulation of E6 particles in close proximity to insulin granules (Figure 4C). The glucose stimulated insulin secretion in E6/91 and E6/12 infected islets was significantly lower than that measured in mock-infected cells (*p* < 0.05, Figure 5).

### 3.2. Echovirus 16 or Echovirus 30 Can Replicate in Human Islets but Not in Human Exocrine Cells

**Figure 6:**
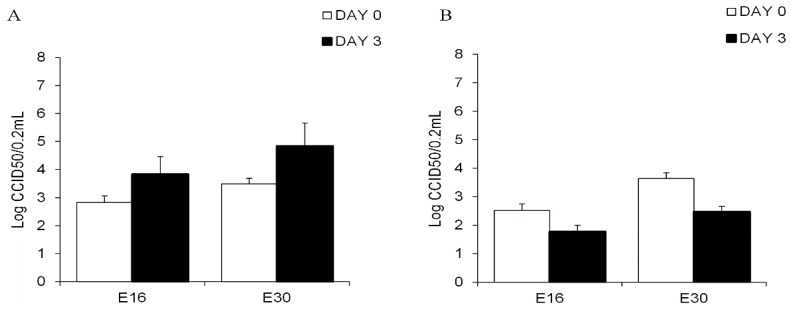
Viral titers of the epidemic strains of E16 and E30 in the culture medium of infected human islets (**A**) and exocrine cells (**B**). Free-floating human exocrine cells from ten donors and islets from seven donors were infected with a 1000 CCID50/0.2 mL of E16 and E30. Aliquots of the culture medium were withdrawn day 0 (empty bar) and day 3 (filled bar). Virus titers were obtained using the CCID50 titration method. Data are presented as log10 (CCID50/0.2 mL), means ± SD from experiment performed in triplicate.

Explanted human islets were productively infected with E16 and E30. The mean CCID50 titers increase in islet after three days of infection with E16 and E30 was 1 logCCID50 and 1.34 logCCID50, respectively (Figure 6A). In contrast, a titer decrease was observed when exocrine cells from all ten donors were inoculated with E16 or E30 (Figure 6B). These viruses did not induce cytopathic effect on exocrine cells for up to five days. Production of HEV capsid protein VP1 could not be detected in the acinar tissue. No structures resembling virus particles or hallmark for virus infection in exocrine cells inoculated with E16 and E30 or mock-infected acinar cells were observed by electron microscopy (data not shown).

### 3.3. Pancreatic Exocrine and Endocrine Cells Express Inflammatory and Antiviral Genes in Response to Echovirus 6 Infection

**Figure 7:**
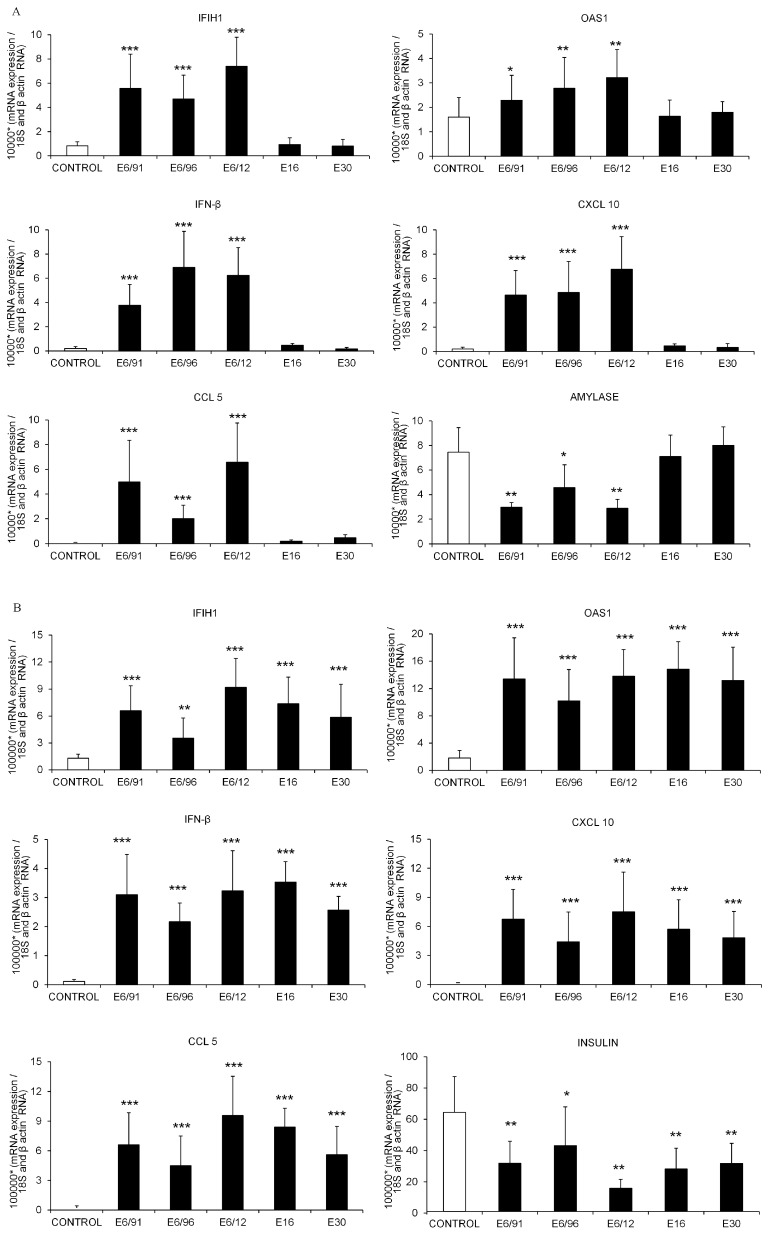
Expression of genes related to virus recognition, antiviral response and amylase in mock-infected and virus-infected pancreatic acinar cells (**A**); Expression of genes related to virus recognition, antiviral response and islet hormone in mock-infected and virus-infected islets (**B**). Isolated human exocrine cell clusters and islets were mock-treated or infected with clinical strains of E6/91, E6/96, E6/12, E16 and E30 for 72 h. mRNA expression of interferon induced with helicase C domain 1 (IF1H1); 2′–5′-oligoadenylate synthetase 1 (OAS1); interferon-β (IFN-β); chemokine (C–X–C motif) ligand 10 (CXCL10); chemokine (C–C motif) ligand 5 (CCL5); amylase and insulin were assayed by real time reverse transcription-polymerase chain reaction (RT-PCR) and normalized by the house keeping gene 18S and β-actin using Δ*C*_t_ method. Gene expression levels are presented as mRNA expression relative to the expression of the housekeeping gene (2^−Δ*C*t^). The expression level of each gene in virus-infected cells was compared to the one measured in mock-infected cells (control) from the same donors. Data are presented as means ± SD and were based on observations from at least three donors. *C*t: cycle threshold. * *p* < 0.05, ** *p* < 0.01, *** *p* < 0.001.

Gene expression was evaluated after infecting pancreatic exocrine cell clusters and islets with E16, E30 and three E6 isolates, representing a cross-section obtained at the beginning (E6/91), mid-stage (E6/96) and at the end (E6/12) of a 21-year endemic circulation of E6 in Cuba. Explanted human acinar cells inoculated with E16 or E30 and mock-infected cells exhibited high amylase gene expression; in contrast, amylase mRNA levels decreased significantly in exocrine cells infected with the three E6 strains (Figure 7A).

High insulin gene expression was observed in mock-infected islets, this expression diminished after inoculation with E6, E16 and E30 (Figure 7B). Notably, E6-infected pancreatic exocrine cell clusters and pancreatic islets showed increased IF1H1, OAS1, IFN-β, CXCL10 and CCL5 mRNA expression in comparison with mock-infected cells (Figure 7A,B). Inoculation with E16 and E30 did not modify the expression of any of the tested genes in exocrine cell clusters (Figure 7A). However, gene expression levels of IF1H1, OAS1, IFN-β, CXCL10 and CCL5 were increased in islets following infection with E16 and E30 compared to the mock-infected control (Figure 7B). There were no differences in the level of expression of these genes between islets infected with E16, E30 or E6 (Figure 7B).

## 4. Discussion

The main finding of this study is that field isolates of E6, sampled over a 21-year lasting period in Cuba, are able to infect both pancreatic exocrine and endocrine cells and induce pro-inflammatory innate immune-responses in both cell types. This is the first report showing that explanted human pancreatic exocrine cells are permissive to E6 infection. In addition, we did not detect any virus replication in pancreatic exocrine cells clusters after infection with the closely related E16 and E30 serotypes, underlying remarkable differences in the tropism to exocrine cell among echovirus serotypes. All seven isolates replicated to high titers and induced a similar degree of cytopathic effect in acinar cells from ten different donors, indicating that no strain-specific variation in the tropism for the exocrine cells clusters was observed for endemic E6 strains.

Human enteroviruses replication in islets and ductal—but not in acinar—cells has been demonstrated in earlier reports [3,18,19,32,33], suggesting that human exocrine pancreas may not represent a target of HEV. Accordingly, we previously showed by virus titration studies, immunoreactivity of HEV VP1 protein and mRNA expression of innate immunity genes that E16 and E30 can productively infect both primary human islets and β cell-derived lines [21,22]. The present study confirms this observation by revealing an increase in viral titer when islets were infected with E16 and E30.

In this study we detected some beta cells characteristically embedded in exocrine cells. This finding has already been reported in previous investigations [19,34] which concluded that, despite the careful handpicking under light microscope, this phenomenon cannot be totally prevented. However, the high viral titers observed at 72 h post-infection in the exocrine fraction indicate that acinar cells, rather than embedded endocrine islets, support a productive E6 infection. Conversely, the viral titers of the islets-tropic E16 and E30 strains decreased in exocrine cells and no VP1-positive cells were found in exocrine acini. Likewise, the expression of the gene encoding amylase, a digestive enzyme marker for acinar cells, significantly decreased in E6-infected exocrine cells in comparison with exocrine cells clusters infected with E16, E30, further supporting the observation that viral replication after E6 infection takes place in the exocrine cells, and did not result just from contaminating β cells.

The presence of immunoreactive HEV capsid protein VP1 in islets and exocrine cells confirmed that both cells types were productively infected by E6. The tropism of the E6 strains towards both human pancreatic islets and acinar cells is also supported by the ultrastructural observation of virus particles in the cytoplasm of both cell types. Additional ultrastructural changes, previously identified in CVB-infected pancreas as “compound membrane-vesicle complexes” [14], were observed both in islets and exocrine acini; these complexes resemble autophagosomes and autophagy-related vesicles and are thought to serve as a scaffold for replication [35]. The finding of accumulation of virus particles in close proximity to insulin granules in E6-infected islets is consistent with reports from ultrastructural analyses of CVB-infected islets and has been hypothesized to display a permissive effect on viral replication [19,36,37].

Moreover, the impairment of insulin secretory ability and the decreased expression of the insulin gene observed after E6 infection additionally supports an effective virus replication within insulin-producing β cells. Remarkably, the presence of insulin granules in the virus-infected beta cells, despite decreased expression of insulin mRNA and a decreased glucose-induced insulin secretion, might suggest that decrease in insulin secretion is due to an impairment of β cell function. While it is clear that E6 replicated extensively in islet β cells, we were unable to determine whether viral replication also occurred in other pancreatic endocrine cells. In any case, we have previously shown that beta cells rather than alfa cells are more likely to support an HEV infection [6,38]. This could be explained by the fact that alfa cells express a vigorous cell-autonomous immune response against viral infection, and thus be able to eradicate the virus more effectively than β cells [39]. 

Remarkably, both primary pancreatic islets and exocrine cells responded to E6 infection by upregulating the transcription of genes involved in viral RNA recognition (IF1H1), antiviral defense (OAS1 and IFN-β) and recruitment of immune cells (CXCL10 and CCL5). Since not all the cells in the clusters are infected at once, it cannot be discarded that the gene expression could be induced in non-infected cells via the release of chemokines or cytokines from infected neighboring cells. This finding not only indicates that acinar cells are well-equipped to recognize and respond to E6 infection, as described for islets, but also suggests that viral replication in acinar cells generates a pro-inflammatory environment potentially attracting immune cells into the pancreas.

This study did not aim to investigate factors potentially influencing the acinar cell tropism of E6 in human pancreas. However, it has been shown that the presence of appropriate virus receptors, the availability of cellular factors essential for virus replication and viral genomic regions capable of interacting with receptor and cellular factors result in more efficient viral infection and progeny production [40,41]. Our data show a serotype-specific difference to replicate and induce damage of acinar cells, which favor the view that distinctive features in echovirus structure might be critical for controlling virus infection. It has been shown that experimentally altered virulence maybe connected to the genetic variations in the region coding for the structural proteins involved in receptor binding. Several studies have indicated that even a single or few amino acid changes in the viral genome can influence the receptor usage and tissue tropism [33,42]. Remarkably, a single amino acid substitution in the capsid protein VP2 could control the expression of the decay-accelerating factor (DAF)-dependent phenotype [43]. It therefore seems likely that virus with different cell tropism could arise during infection in humans, possibly driven by the host immune response. Our study is important in this regard and has generated results that warrant further scrutiny. Further investigations are, therefore, required in order to elucidate determinants of E6 tropism toward endocrine and exocrine pancreas. It remains to be established whether other serotypes of echovirus than E6 also infect pancreatic exocrine cells. 

Morphological changes in the acinar tissue, along with mild to moderate exocrine pancreatic insufficiency, were shown to represent early events in type 1 diabetes disease process [44,45,46,47]; notably, inflammatory processes have been detected in exocrine pancreas from patients with type 1 diabetes [48,49]. Furthermore, it has been recently hypothesized that β cell autoantibodies result from injuries to the exocrine pancreatic portion [50]. However, whether exocrine dysfunction in type 1 diabetes represents a primary damage induced by the same pathogenic event that led to islets destruction and beta cells loss, remains an unanswered question [51,52]. Our results suggest that the infection with HEV strains targeting pancreatic islets and exocrine cells may act as a common environmental trigger for endocrine and exocrine pancreatic dysfunction in patients with type 1 diabetes. It is plausible that the infection of exocrine cells represents the conduit for islets infection. Therefore, the acinar cells infection and the consequent innate immune-response activation may explain the pancreatic inflammation, which sets the stage for the development of autoimmunity in patients with type 1 diabetes [50,53].

It should be noted that although strains of E6 capable of damaging and inducing pro-inflammatory responses in pancreas have been endemic in Cuba over the last 21 years, the incidence of type 1 diabetes in this area has remained low and relatively constant in this timeframe [24,54,55]. In Finland, the country with the highest incidence of type 1 diabetes in the world, molecular epidemiological and evolutionary analysis of Finnish E6 strains revealed that this virus is also endemic in Finland [56]. Such a divergent scenario further highlights the complexity of tracing HEV-disease association in type 1 diabetes and other pancreas diseases.

In conclusions, this study demonstrates that field isolates of E6 infect both the exocrine and endocrine pancreatic cells, whereas E16 and E30 strains have selective tropism for endocrine cells. Thus, it could be argued that while endocrine cells are permissible to a wide range of HEV serotypes, human pancreatic exocrine cells support productive infection of certain HEV serotypes. The determinants underlying the tropism of E6 for human exocrine and endocrine cells, and whether there is a link with the development of pancreatitis and type 1 diabetes in humans, warrant further investigations.
